# Antioxidant agents against trichothecenes: new hints for oxidative stress treatment

**DOI:** 10.18632/oncotarget.22800

**Published:** 2017-11-30

**Authors:** Qinghua Wu, Xu Wang, Eugenie Nepovimova, Yun Wang, Hualin Yang, Li Li, Xiujuan Zhang, Kamil Kuca

**Affiliations:** ^1^ College of Life Science, Institute of Biomedicine, Yangtze University, Jingzhou 434025, China; ^2^ Department of Chemistry, Faculty of Science, University of Hradec Kralove, Hradec Kralove 50003, Czech Republic; ^3^ National Reference Laboratory of Veterinary Drug Residues (HZAU) and MAO Key Laboratory for Detection of Veterinary Drug Residues, Huazhong Agricultural University, Wuhan 430070, China; ^4^ College of Horticulture and Gardening, Yangtze University, Jingzhou 434025, China

**Keywords:** trichothecenes, T-2 toxin, deoxynivalenol, oxidative stress, antioxidant agents

## Abstract

Trichothecenes are a group of mycotoxins mainly produced by fungi of genus *Fusarium*. Due to high toxicity and widespread dissemination, T-2 toxin and deoxynivalenol (DON) are considered to be the most important compounds of this class. Trichothecenes generate free radicals, including reactive oxygen species (ROS), which induce lipid peroxidation, decrease levels of antioxidant enzymes, and ultimately lead to apoptosis. Consequently, oxidative stress is an active area of research on the toxic mechanisms of trichothecenes, and identification of antioxidant agents that could be used against trichothecenes is crucial for human health. Numerous natural compounds have been analyzed and have shown to function very effectively as antioxidants against trichothecenes. In this review, we summarize the molecular mechanisms underlying oxidative stress induced by these compounds, and discuss current knowledge regarding such antioxidant agents as vitamins, quercetin, selenium, glucomannan, nucleotides, antimicrobial peptides, bacteria, polyunsaturated fatty acids, oligosaccharides, and plant extracts. These products inhibit trichothecene-induced oxidative stress by (1) inhibiting ROS generation and induced DNA damage and lipid peroxidation; (2) increasing antioxidant enzyme activity; (3) blocking the MAPK and NF-κB signaling pathways; (4) inhibiting caspase activity and apoptosis; (5) protecting mitochondria; and (6) regulating anti-inflammatory actions. Finally, we summarize some decontamination methods, including bacterial and yeast biotransformation and degradation, as well as mycotoxin-binding agents. This review provides a comprehensive overview of antioxidant agents against trichothecenes and casts new light on the attenuation of oxidative stress.

## INTRODUCTION

**Figure 1:**
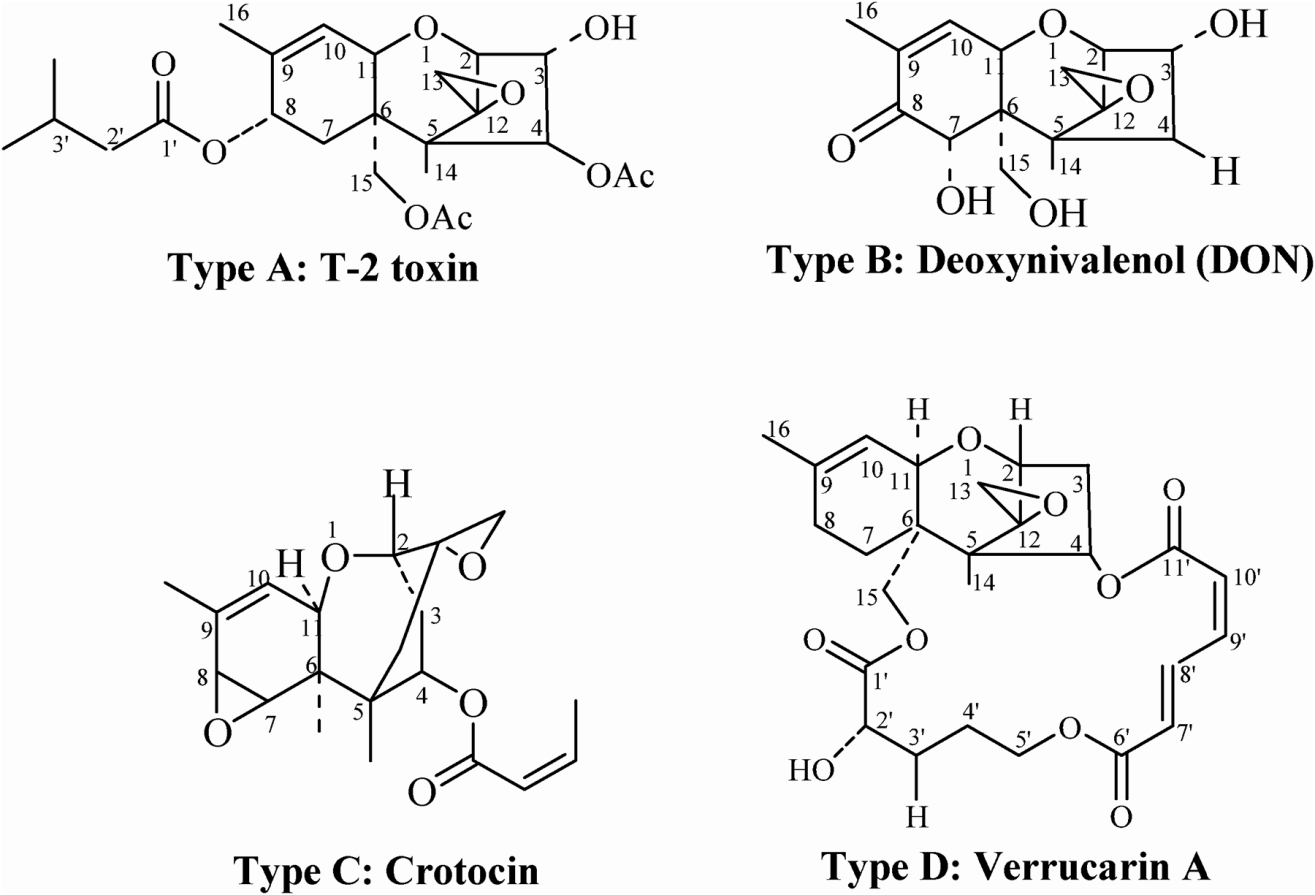
Chemical structure of trichothecenes (Types A-D)

Trichothecenes, a large group of chemically related mycotoxins mainly produced by fungi of genus *Fusarium*, are among the most commonly occurring contaminants in the food chain. Trichothecenes are divided into four types (A–D) according to their characteristic functional groups. Types A and B are of greater concern to people due to their high toxicity and frequent presence as food contaminants [1, 2]. For both of these reasons, T-2 toxin and deoxynivalenol (DON), which belong to type A and type B, respectively, are considered to be the most important compounds of this class (Figure 1) [3–5]. The most toxic trichothecene, T-2 toxin, causes growth inhibition and sublethal or even lethal toxicosis in humans and farm animals. Alimentary toxic aleukia (ATA), a fatal human disease, is primarily associated with T-2 toxin [6, 7]. In Europe, especially in the Nordic countries, contamination of cereals with T-2 toxin is a serious problem [8–11]. In aerosol form, T-2 toxin can easily penetrate organisms through the lungs. Reactions of the skin or intestinal mucosa are provoked rapidly after direct contact through dermal application or ingestion of the compound. In addition, T-2 toxin is a radiomimetic compound, and thus exacerbates the effect of ionizing radiation [12–14].

**Figure 2:**
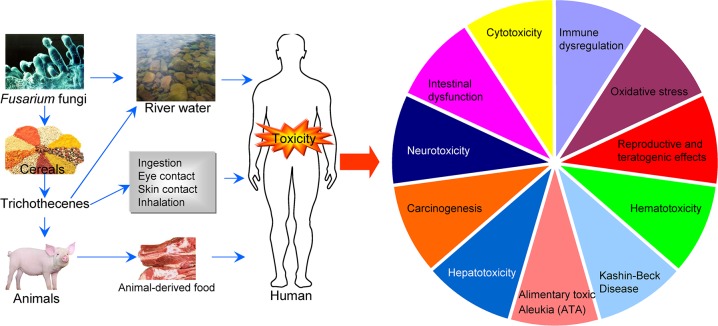
Routes of exposure and various toxicities of trichothecenes

DON is less toxic than T-2 toxin and many other trichothecenes, but it is the most widely distributed trichothecene and is commonly found in barley, corn, wheat, and mixed feed [4, 5, 15]. At the molecular level, DON binds to the ribosome and induces ribotoxic stress, leading to the activation of mitogen-activated protein kinases (MAPK), cell-cycle arrest, and apoptosis [16]. The toxic effects of DON include emesis and anorexia, alteration of intestinal and immune functions, reduced absorption of nutrients, and elevated susceptibility to infection and chronic diseases [17]. Importantly, DON contamination in wheat flour may be involved in the fluctuating but high prevalence of Kashin-Beck disease (KBD) [18]. DON contamination has also been reported in countries including the Czech Republic, Italy, Spain, Croatia, Canada, and Germany [19–24]. The routes of exposure and various toxicities of trichothecenes are illustrated in Figure 2.

The evidence accumulated to date shows that oxidative stress is an important toxic mechanism of trichothecenes [25–29], which significantly increase levels of reactive oxygen species (ROS) and deplete intracellular reduced glutathione (GSH). Moreover, these compounds increase lipid peroxidation, leading to single-strand breaks in DNA [30–31]. In addition, they activate signaling pathways including MAPK, JAK/STAT, and NF-κB, which trigger apoptosis [4, 5, 32, 33]. Trichothecenes activate MAPK by a mechanism called the “ribotoxic stress response” (RSR), which drives both cytokine gene expression and apoptosis in macrophages [34]. Over the last decade, mitochondria-related toxic mechanisms became an active area of research in this field. In addition to ribosomes, mitochondria are considered to be targets of trichothecenes [35, 36]. Importantly, mitochondrial dysfunction caused by trichothecenes is largely associated with oxidative stress. Normally, trichothecenes damage the normal function of mitochondria and release free radicals (including ROS), induce lipid peroxidation, and change the antioxidant status of cells, thereby reducing the activity of antioxidant enzymes [4, 5]. Thus, oxidative stress mediated by trichothecenes and their related toxicities pose a great risk to human health.

As trichothecenes cause oxidative stress, researchers are exploring potential antioxidant agents for use against trichothecenes. To date, many natural compounds with antioxidant activity against these compounds have been identified. For example, the traditional antioxidant agents vitamins A, C, and E exhibit beneficial effects against DON-induced protein and lipid peroxidation [37]. Quercetin can reduce apoptosis caused by T-2 toxin [38]. Selenium can prevent erythrocyte membrane damage induced by T-2 toxin [39]. N-Acetyl-cysteine (NAC) protects chicken growth plate chondrocytes from T-2 toxin-induced oxidative stress [40]. In addition, antimicrobial peptides can improve feed efficiency, immune function, and anti-oxidative capacity, as well as alleviate organ damage caused by DON [41]. Moreover, some plant extracts, including polyphenol epigallocatechin 3-gallate (EGCG), leontopodic acid (LA), and quince seed mucilage, have marked antioxidant effects against trichothecenes. Therefore, elucidation of the anti-oxidative activities of various compounds is crucial for the detoxification of trichothecenes. Previously, however, no review article had fully summarized the data regarding such antioxidant agents.

Therefore, in this review, we will first briefly discuss the oxidative stress mechanisms of trichothecenes. Our main goal, however, is to explore our current knowledge regarding potential antioxidant agents against trichothecenes, as well as their antioxidant mechanisms. We focus specifically on the most toxic and widespread trichothecenes, T-2 toxin and DON. Finally, we will discuss some decontamination methods, including bacterial and yeast biotransformation and degradation, as well as mycotoxin-binding agents. We believe that this review provides a comprehensive overview of current knowledge on detoxification of trichothecenes and casts new light on attenuation of oxidative stress.

## OXIDATIVE STRESS AND TRICHOTHECENES

Oxidative stress is an important mechanism of trichothecene toxicity. Normally, trichothecenes disrupt the normal function of mitochondria and generate free radicals, including ROS. These oxidative compounds induce lipid peroxidation and change the antioxidant status of the cells, thereby reducing the activity of antioxidant enzymes such as glutathione-S-transferase (GST), superoxide dismutase (SOD), and catalase (CAT) in the body [4]. DNA damage is an early event associated with the generation of ROS and lipid peroxidation. Some signaling pathways, including MAPK, JAK/STAT, and NF-κB, are subsequently induced by oxidative stress, and the caspase-mediated apoptosis pathways are also activated [34].

Oxidative stress is the mechanism by which T-2 toxin causes DNA damage and apoptosis [30, 31, 42]. Indeed, T-2 toxin can induce generation of ROS as early as 30 min after exposure, followed by significant depletion of glutathione levels and elevated lipid peroxidation [30]. Moreover, the ROS-mediated mitochondrial pathway plays an important role in T-2 toxin-induced apoptosis [29, 43]. In granulosa cells and murine embryonic stem cells, T-2 toxin treatment induces ROS accumulation, resulting in reduction of mitochondrial transmembrane potential. In addition, T-2 toxin strongly inhibits the mitochondrial electron transport system (ETS) in rat cardiomyocytes [44]. In murine embryonic stem cells (mESCs), T-2 toxin inhibited mitochondrial biogenesis by increasing ROS levels, leading in turn to inhibition of ESC differentiation [45]. In addition, proteomic changes in chicken primary hepatocytes exposed to T-2 toxin are associated with oxidative stress and mitochondrial enhancement [46]. More recently, our group found that nitric oxide (NO)-mediated mitochondrial damage plays a critical role in T-2 toxin-induced apoptosis and growth hormone deficiency in rat anterior pituitary GH3 cells [7]. We found that T-2 toxin caused significant increases in NO generation, cell apoptosis, iNOS activity, production of inflammatory factors, and caspase pathway activation, while also decreasing growth hormone (GH) production and mitochondrial membrane potential (ΔΨm). These data show that mitochondria are a primary target of T-2 toxin-induced NO, and NO is a key mediator of T-2 toxin-induced apoptosis and GH deficiency via a mitochondria-dependent pathway.

T-2 induces oxidative damage in the liver [46]. Specifically, T-2 toxin exposure causes significant oxidative damage by depleting hepatic glutathione, increasing lipid peroxidation, altering the activity of antioxidant enzymes, and inducing protein oxidation in a time-dependent manner. Moreover, the oxidative stress and apoptosis induced by T-2 toxin are involved in the developmental toxicity of this compound in zebrafish embryos [47]. We also know that T-2 toxin can cross the blood brain barrier [48]. Consistent with this, percutaneously or subcutaneously applied T-2 toxin causes oxidative damage in the brain. Mice treated with T-2 toxin via either exposure route exhibit a time-dependent increase in ROS generation, glutathione depletion, lipid peroxidation, and protein carbonyl content in the brain [27].

The oxidative damage caused by T-2 toxin is involved in the mechanism of KBD [49]. The increase in thiobarbituric acid-reactive substances (TBARS) and decrease in antioxidant levels following T-2 toxin treatment may induce oxidative stress in joint tissues and contribute to the pathological process of cartilage damage in KBD. In addition, in aquatic species, T-2 toxin induces anemia and oxidative stress, as well as altering the immune response [50, 51]. Shrimp exposed to T-2 toxin in the diet showed elevated ROS levels, SOD activity, and histopathological changes in the hepatopancreas [51].

Oxidative stress is also an important toxic mechanism of DON, a type B trichothecene. The manifestations of this stress are elevated levels of ROS and malondialdehyde (MDA), and reduced levels of GSH and SOD. In addition, as T-2 toxin, DON can also cause mitochondrial damage by decreasing mitochondrial membrane potential and inducing apoptosis accompanied by upregulation of apoptosis-related factors including caspase-3, caspase-8, and caspase-9 [36, 52]. DON induces apoptosis in splenic lymphocytes and PC12 cells via a ROS-mediated mitochondrial pathway [53, 54]. Yang et al. (2014) found that DON triggered potential genotoxicity in human peripheral blood lymphocytes via oxidative damage [55]. Furthermore, those authors showed that DON exposure increased peroxidation, decreased antioxidant activity, and inhibited DNA repair and expression of heme oxygenase-1 (HO-1).

Thus, it is clear that oxidative stress plays important roles in the toxicity of trichothecenes. Interestingly, some cells can mount their own antioxidant defenses following exposure to these toxins. For example, when HepG2 cells are exposed to DON, ROS levels are significantly elevated, but as time passes, antioxidant enzymes such as SOD and CAT are highly upregulated, presumably reflecting the cells’ effort to detoxify the damage caused by DON [56]. Thus, the antioxidant defense system of HepG2 cells represents an insufficient adaptation aimed at minimizing DON-induced oxidative injury. In addition, JunD, a member of the AP-1 family of transcription factors, plays an important role in defense against oxidative stress induced by T-2 toxin [57]. A similar self-defense capacity has also been observed in *in vivo* studies. Short-term oral exposure of T-2 toxin initiates lipid oxidation in chicken liver, but the antioxidant defense system eliminates the free radicals and inhibits the oxidative stress [58].

**Figure 3:**
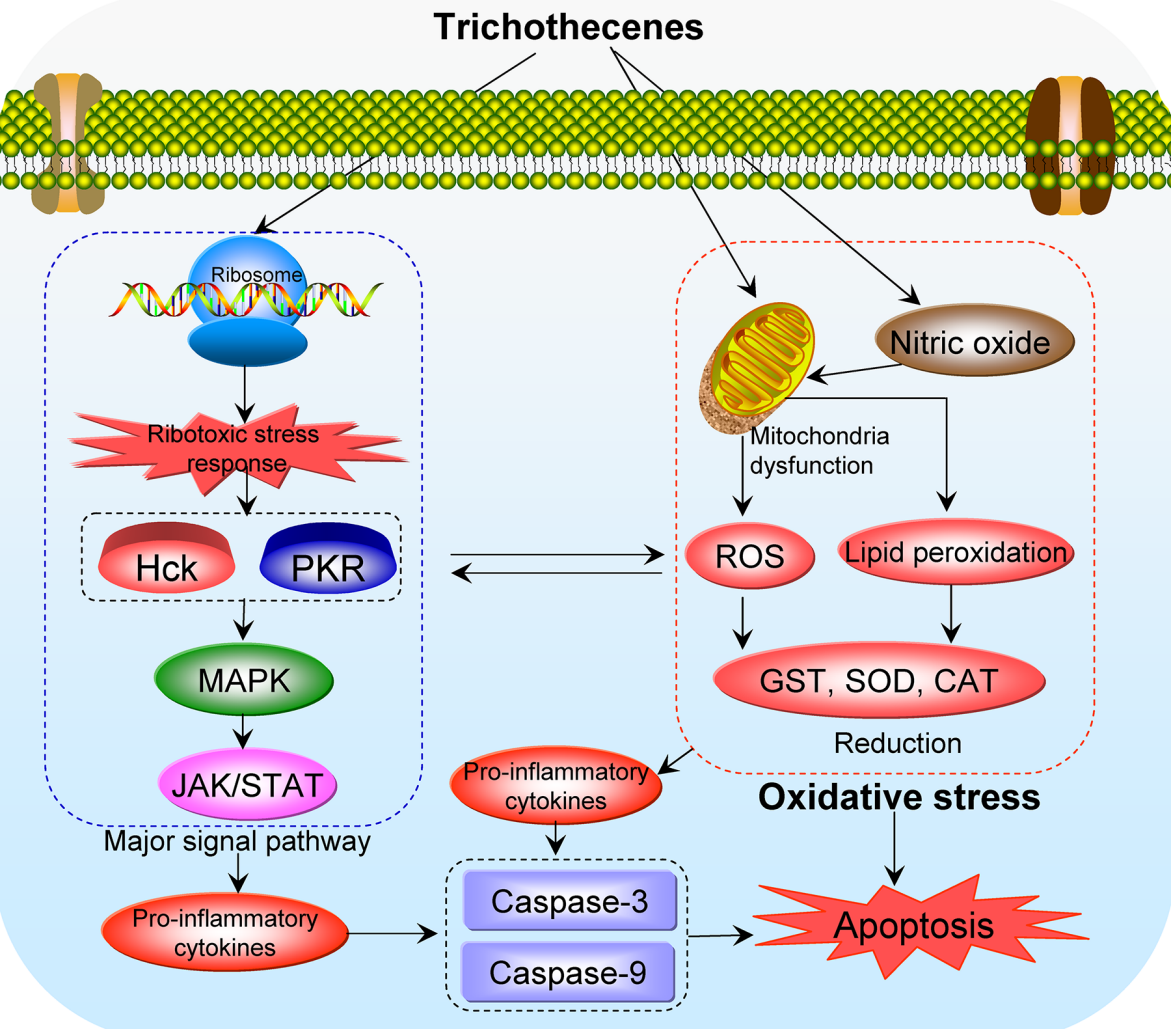
Proposed mechanisms of oxidative stress-mediated toxicity of trichothecenes

Collectively, these findings show that oxidative stress is an important underlying toxic mechanism of trichothecenes. Trichothecenes generate ROS that induce lipid peroxidation, which in turn leads to changes in membrane integrity, cellular redox signaling, and cellular antioxidant status. DNA damage correlates with, but precedes, the generation of ROS and lipid peroxidation. Because trichothecenes are commonly found in food and feed, the cellular effects of these toxins in relation to oxidative stress, as well as effective measures for combating their toxicity, should be addressed in future studies. The proposed mechanisms underlying oxidative stress induced by trichothecenes are shown in Figure 3.

## ANTIOXIDANT AGENTS AGAINST TRICHOTHECENES

As discussed above, oxidative stress plays key roles in the toxicity of trichothecenes. Oxidative damage caused by trichothecenes is one of the mechanisms underlying toxin-induced cell injury and DNA damage, which eventually lead to tumorigenesis [4]. Currently, researchers are trying to identify potential agents that effectively prevent trichothecene-induced oxidative stress and the associated immunotoxicity. Interestingly, agents such as vitamins, quercetin, selenium, glucomannan, amino acids, nucleotides, antimicrobial peptides, bacteria, polyunsaturated fatty acids, oligosaccharides, and some plant extracts exert promising anti-oxidative effect against trichothecenes. Accordingly, these compounds have attracted close attention from researchers. The various antioxidant agents with potential for use against trichothecenes are presented in Table 1.

**Table 1:** Summary of the antioxidant agents against trichothecenes

Entry	Agents	Cell or animal model	Specific effects	References
1	Vitamins E, A, C	Murine YAC-1lymphoblastic cell	Against the DON-induced protein and lipid peroxidation; increased the cell viability and cell proliferation.	[37]
2	Vitamins C, E	Rat	Decrease the lipid peroxides and TBARS values caused by T-2 and DON; decreased mortality.	[59]
3	Coenzyme Q10, Vitamin E	Mice	Decreased DNA damage caused by T-2 toxin; Protection against cell death and glutathione depletion caused by T-2 toxin.	[61]
4	Lutein	HT-29 cell	Decreased DON-induced oxidative stress and apoptosis.	[26]
5	Quercetin	Rabbit ovary	Increased cell proliferation and reduced cell apoptosis caused by T-2 toxin.	[38]
6	Quercetin	Porcine ovarian granulosa cell	Effectived in maintaining and increasing of TAS, activitied SOD and glutathione peroxidase (GPx).	[62]
7	Selenium	Mice	T-2 toxin decreased the number of CD8^+^, CD3^+^, CD4^+^ T-lymphocytes, whereas Se mitigated these decreases.	[65]
8	Selenium	Mice	Prevented erythrocyte membrane damage induced by T-2 toxin.	[39]
9	Selenium chondroitin sulfate nanoparticles (SeCS)	KBD patients	Blocked the T-2 toxin-induced chondrocyte apoptosis by decreasing the expression of ATF2, JNK and p38.	[69]
10	Modified glucomannan	Chicken	Made a 45% reduction of lipid peroxidation in the liver in comparison to the effects of T-2 toxin alone.	[74]
11	Glucomannan	Pig	Restored anti-ovalbumin immunoglobulin G production, which was significantly reduced in pigs expose to T-2 toxin.	[72]
12	N-acetyl-cysteine (NAC)	Chicken	Decreased the levels of ROS and MDA which were increased by T-2 toxin.	[77]
13	Arginine, Glutamine	Pig	Alleviated the impairment induced by DON stress and immune relevant cytokines in growing pigs.	[78]
14	Glutamic acid	Pig	Increased the plasma activities of SOD and GSH-Px and the proliferating cell nuclear antigen (PCNA) labeling indexes for the jejunum and ileum.	[79]
15	L-carnitine	Rat	Reduced toxicity and prevented the hepatocytes from abnormal caspase-3 activity and apoptosis caused by T-2 toxin.	[81]
16	Nucleotides	Chicken	Reduced the extent of DNA damage induced by T-2 toxin in leukocytes.	[82]
17	Composite antimicrobial peptides (CAP)	Pig	DON decreased peripheral lymphocyte proliferation, whereas supplementation with CAP increased it on day 15 and 30.	[41]
18	Composite antimicrobial peptides (CAP)	Pig	Improved intestinal morphology and promoted intestinal epithelial cell proliferation and protein synthesis.	[84]
19	Epigallocatechin-3-gallate (EGCG)	HT-29 cell	Protected against DON mediated-oxidative stress, up regulation of NF-κB, COX-2 and caspase-3 activated apoptosis.	[85]
20	Leontopodic acid (LA)	U937 cells	Protected cells from DON-induced cell damage; enhanced glutathione peroxidase activity.	[87]
21	Quince seed	Rabbit	Showed more and better healing effects on dermal toxicity caused by T-2 toxin.	[88]
22	Rutin	Rat	Reversed the T-2 toxin-mediated increase of lipid peroxidation parameter, SOD and glutathione concentration in liver homogenate.	[91]
23	lycopene	Chicken	Inhibited T-2 toxin induce oxidative stress and protected the cellular level of GSH	[92]
24	Lactobacillus plantarum JM113	Chicken	Reduced MDA activity in the jejunal mucosa; increased the mRNA levels of Nrf2 and its corresponding downstream HO-1 gene.	[93]
25	Lactobacillus rhamnosus GG (LGG)	Mice	Prevented or treated the unwanted adverse effects of DON/ZEA in mice by regulation of goblet cell mucus secretion, improvement of plasma D-lactate, IL-8/CXCL8 and serum Ig levels.	[94]
26	PUFA, DHA, EPA	Mice	Suppressed DON-induced IgAN.	[95]
27	Docosahexaenoic acid (DHA)	Mice	Suppressed the PKR and CREB kinase pathways thus inhibited the IL-6 transcription	[97]
28	Galacto-oligosaccharides (GOSs)	Caco-2 Cell Monolayers; B6C3F1 Mice	Prevented the DON-induced loss of epithelial barrier function; stabilized the expression and cellular distribution of claudin3 and suppressed; In mice, GOSs prevented the DON-induced mRNA overexpression of claudin3 and CXCL8 homolog keratinocyte hemoattractant.	[98]

### Vitamins

During the last decade, antioxidant agents such as vitamins and microelements have received a great deal of attention. One earlier study demonstrated that dietary use of vitamins E and C can decrease lipid peroxide and TBARS levels, thereby protecting against acute toxicosis caused by trichothecenes (Entry 1–4, Table 1) [59]. In addition, Strasser et al. (2013) [37] further demonstrated that the antioxidant vitamins E, A, and C had beneficial effects against DON-induced protein and lipid peroxidation. These compounds significantly increased cell viability and cell proliferation following toxin exposure. Lutein, a non-vitamin A carotenoid, has been implicated in maintaining eye health, specifically in regard to age-related macular degeneration [60]. The cytoprotective effect of lutein against DON-induced oxidative stress was tested in HT-29 cells [26]. In that study, pretreatment of cells with 10 μM lutein resulted in 95% cell viability. Moreover, lutein combated DON-induced oxidative stress and downregulated expression of inflammatory genes such as NF-κB and COX-2. Lutein also prevented DON-induced migration of NF-κB into the nucleus. Morphological studies revealed that lutein prevented DON-induced apoptosis. In addition to the vitamins, coenzyme Q_10_ decreases T-2 toxin-induced DNA damage in mouse liver [61].

### Quercetin

Quercetin, a member of the large family of flavonoid compounds that exert manifold biological effects, has anti-inflammatory, antioxidant, free radical-scavenging, and metal-chelating properties. Quercetin increases rabbit ovary cell proliferation and diminishes the apoptosis caused by T-2 toxin [38]. Recent work showed that quercetin has no effect in the elimination of ROS generation induced by T-2 toxin, but is still effective in maintaining and increasing total antioxidant status (TAS) and the activities of SOD and glutathione peroxidase (GPx) in porcine granulosa cells *in vitro* [62]. Thus, based on the *in vitro* studies, quercetin has the potential to inhibit T-2 toxin-induced oxidative stress and apoptosis; however, additional *in vivo* studies are needed to verify this conclusion (Entry 5-6, Table 1).

### Selenium

Selenium is an essential micronutrient that exerts multiple and complex effects on human health. This element is important for human well-being largely due to its potent antioxidant, anti-inflammatory, and antiviral properties (Entry 7-9, Table 1) [63]. Selenium participates in protection of cells against excess H_2_O_2_, heavy metal detoxification, and regulation of the immune and reproductive systems [64]. In the context of mycotoxins, selenium can prevent erythrocyte membrane damage induced by T-2 toxin [39]. The protective effect of selenium may be due to its membrane-stabilizing properties. Salimian et al. (2014) [65] also found that selenium could exert a marked effect against the immunotoxic effects of T-2 toxin in T lymphocytes. KBD is an endemic osteochondropathy manifested by chondrocyte necrosis and apoptosis, cartilage degeneration, and matrix degradation [66], and T-2 toxin is one of the important etiological factors for this disease [67]. In addition, selenium deficiency has also been proposed as a risk factor for KBD. In this view, compromised selenoprotein function leads to oxidative stress and apoptosis, which ultimately manifests as KBD [68]. Recently, selenium chondroitin sulfate nanoparticles (SeCS) with a size range of 30–200 nm were obtained by the research group of Guo et al., who investigated the inhibitory effects of SeCS on T-2 toxin-induced apoptosis of chondrocyte from KBD patients [69]. The results revealed that SeCS partly blocked T-2 toxin-induced chondrocyte apoptosis by decreasing the activity of ATF2, JNK, and p38. Thus, SeCS could be used for prevention and treatment of KBD, as well as other Se-deficiency diseases.

### Glucomannan

Glucomannan is a soluble, fermentable, and highly viscous dietary fiber derived from the root of the elephant yam or konjac plant, which is native to Asia [70]. Because ingestion of this compound promotes human health, over the last two decades it has become more frequently used in western countries [71]. During that time, researchers also studied the antioxidant role of glucomannan against trichothecenes (Entry 10-11, Table 1). In pigs [72], glucomannan dietary supplementation exerted no deleterious effects but protected against T-2 toxin immunotoxicity during a vaccine protocol. In another study [73], a modified glucomannan mycotoxin-adsorbing agent was shown to counteract reduced weight gain in T-2 toxin-exposed pigs. This modified glucomannan also reduced the number of *Salmonella typhimurium* bacteria in the cecum and cecal contents of T-2 toxin-exposed pigs. In addition, the antioxidant effects of organic selenium (Sel-Plex™) and modified glucomannan (Mycosorb™) against T-2 toxin have been assessed [74]. Inclusion of modified glucomannans into a T-2 toxin-contaminated diet provides partial protection against the detrimental effects of the toxin on antioxidant defenses in chicken liver [61], and the combination of modified glucomannans with organic selenium confers further protection against toxin-induced antioxidant depletion and lipid peroxidation in that tissue.

### Amino acids

Amino acids play important roles in digestion and absorption of dietary nutrients, metabolism of glucose and lipids, acid–base balance, anti-oxidative responses, detoxification of xenobiotics, and immunity [75]. Dietary antioxidants and their roles in preventing mycotoxin toxicity have attracted increasing attention in recent years in studies aimed at evaluating the potential benefits of amino acids in the diet (Entry 12-15, Table 1). NAC is an effective source of sulfhydryl groups in cells and a scavenger of free radicals that may interact with ROS such as OH· and H_2_O_2_ [76]. Recently, NAC was shown to protect chicken growth plate chondrocytes from T-2 toxin-induced oxidative stress [77]; specifically, NAC significantly decreased ROS and MDA levels induced by toxin exposure. Moreover, a greater increase in CAT and SOD activity was observed. Thus, NAC may confer a therapeutic benefit against metaphyseal chondrodysplasia by improving the antioxidant capacity of growth plate chondrocytes. Similarly, supplementation with glutamic acid in piglets exerts a strong anti-oxidative effect against DON. A series of studies [78, 79] showed that DON significantly induces oxidative stress in piglets, but this stress is remarkably reduced by glutamic acid supplementation, as reflected by changes in oxidative parameters in blood and tissues. Meanwhile, DON causes obvious intestinal injury, as determined by microscopic observations and the use of permeability indicators, but this damage is alleviated by glutamic acid supplementation. Moreover, the inhibitory effect of DON on the Akt/mTOR/4EBP1 signaling pathway is reduced by glutamic acid supplementation [79]. Addition of glutamic acid to DON-treated cells increases plasma activities of SOD and glutathione peroxidase (GSH-Px) and the proliferating cell nuclear antigen (PCNA) labeling indexes in jejunum and ileum. These findings indicate that glutamic acid has the potential to repair injuries associated with oxidative stress, intestinal injury, and signaling inhibition.

Arginine and glutamine also play important roles in pig nutrition. The effects of dietary supplementation with arginine and glutamine on both the impairments induced by DON stress and immune cytokines in growing pigs have been studied [78]. The results of that work revealed that levels of IGF1, GH, and SOD in the amino acid-treated groups were significantly higher than those in the toxin control. Moreover, the IL-2 and TNF-α values in the amino acid-treated groups were similar to those in the non-toxin control, and significantly lower than those in the toxin control. Thus, dietary supplementation with arginine and glutamine can alleviate the impairments induced by DON stress and immune cytokines in growing pigs.

L-carnitine not only diminishes oxidative stress but also protects mitochondria against fatty acid stress and apoptosis by inhibiting mitochondrial swelling and Cyt-c release [80]. Pretreatment of rats with L-carnitine prevents hepatocytes from expressing abnormal caspase-3 activity and undergoing apoptosis in response to T-2 toxin [81]. Thus, due to its mitochondrial protective effects, L-carnitine supplementation represents a promising method for diminishing or preventing the toxicity induced by T-2 toxin.

### Nucleotides

Nucleotides are “semi-” or “conditionally” essential nutrients, but they may become essential in pathological conditions that demand intense nucleic acid and protein synthesis. Evidence has shown that dietary nucleotides have the potential to reduce the extent of DNA damage induced by T-2 toxin in immune cells [82]. In a study by Frankic et al. (2006) [82], T-2 toxin induced DNA fragmentation in chicken spleen leukocytes, but supplementation with nucleotides reduced the amount of damage (Entry 16, Table 1). This finding highlights the possible beneficial effect of dietary nucleotides on the immune system in cases of mycotoxin intoxication.

### Antimicrobial peptides

Antimicrobial peptides are small cationic molecules that are part of the nonspecific defense system; these compounds kill bacteria, modulate bacterial infections, and coordinate host responses to infection [83]. Antimicrobial peptides have the capacity to improve feed efficiency, immune function, and anti-oxidant capacity, and alleviate organ damage caused by DON, and thus have a protective effect in piglets challenged with this toxin [41]. In addition, composite antimicrobial peptides (CAPs) can repair intestinal injury induced by DON; specifically, CAPs improve intestinal morphology and promote intestinal epithelial cell proliferation and protein synthesis (Entry 17-18, Table 1) [84].

### Plant extracts

Many natural antioxidants, which provide multiple health benefits, are present in tea, a common beverage (Entry 19–23, Table 1). In a recent study of the cytoprotective effect of green tea on DON-induced toxicity in HT-29 cells [85], EGCG prevented DON-induced cytotoxicity to HT-29 cells in a dose-dependent manner. Even the lowest concentration (5 μM) of EGCG protected against the highest concentration of DON tested, and pretreatment with 20 μM EGCG yielded 99% cell viability. EGCG also protected against oxidative stress, upregulation of NF-κB, COX-2, and caspase-3 activated apoptosis. These results suggest that EGCG acts as cytoprotective agent against DON-induced toxicity. Therefore, the use of EGCG represents an attractive strategy for cytoprotection of cells against the actions of trichothecenes.

LA is a fully substituted hexaric acid derivative obtained from *Leontopodium alpinum Cass.*, commonly known as edelweiss [86]. This molecule exerts a strong antioxidant capacity, and in particular was shown to protect U937 cells from DON-induced oxidative damage and increase GPx activity in these cells [87]. The increase in the activity of detoxifying enzymes is probably the main mechanism underlying antioxidant-mediated chemoprevention.

Quince seed mucilage exerts healing effects on dermal toxicity caused by T-2 toxin [88]. Materials obtained from *Cydonia oblonga* species (including quince seed mucilage) are an excellent natural source of phenolic acids and flavonoids, which are considered potent antioxidants [89, 90]. Quince seed mucilage may diminish the dermal toxicity of T-2 toxin via its antioxidant properties. Similarly, rutin exhibits strong anti-oxidative capacity against T-2 toxin in rat liver, and decreases T-2 toxin-induced lipid peroxidation [91]. In addition, lycopene from fresh tomatoes prevents T-2 toxin-induced oxidative stress and helps to maintain the cellular level of GSH [92].

### Bacteria

Bacteria exhibit promising antioxidant activities against trichothecenes (Entry 24-25, Table 1). A very recent study investigated the antioxidant capacity of *Lactobacillus plantarum* JM113 isolated from healthy intestinal contents of Tibetan chicken and its protective effect on broiler chickens challenged with DON [93]. The results revealed that dietary supplementation with DON decreased SOD activity in serum and increased MDA in the jejunal mucosa. However, supplementation with *L. plantarum* JM113 to a DON-contaminated diet caused a significant reduction in MDA activity in the jejunal mucosa. Moreover, DON decreased the expression of nuclear factor erythroid 2-related factor 2 (Nrf2), whereas Nrf2 mRNA levels and expression of the downstream gene HO-1 increased following *L. plantarum* JM113 treatment. Thus, *L. plantarum* JM113 has high antioxidant activity, and supplementation of this compound in feed protected the integrity of the intestinal barrier in broilers challenged with DON, suggesting that it could be used to alleviate the negative effects of DON in poultry. Another study assessed the ability of *L. rhamnosus* GG (LGG) to improve intestinal barrier functions and ameliorate inflammation in Balb/c mice fed with DON-containing diets [94]. The results showed that LGG partially prevented or reversed the adverse effects of DON in mice by regulating goblet cell mucus secretion and normalizing plasma D-lactate, IL-8/CXCL8, and serum Ig levels. Thus, LGG offers a potential dietary intervention strategy against intestinal exposure to mycotoxins.

### Polyunsaturated fatty acid

Consumption of DON induces IL-6–dependent IgA nephropathy (IgAN) in mice [95]. Consumption of the n-3 polyunsaturated fatty acid (PUFA), docosahexaenoic acid (DHA), or eicosapentaenoic acid (EPA) suppresses DON-induced IgAN in mice, in agreement with the proposed anti-inflammatory action of these fatty acids [95]. Moreover, these results are consistent with randomized clinical trials demonstrating that fish oil consumption retards loss of renal function in IgAN patients [96]. In a subsequent study [97], DHA was shown to suppress the RNA-activated protein kinase R (PKR) and cAMP response element-binding protein (CREB) kinase pathways, thereby inhibiting the IL-6 transcription. Therefore, suppression of IL-6 expression by DHA might have general importance in human health, specifically in regard to the prevention and treatment of inflammatory and autoimmune diseases mediated by this pro-inflammatory cytokine (Entry 26–27, Table 1).

### Oligosaccharide

Galacto-oligosaccharides (GOS) are beneficial food additives capable of protecting vulnerable segments of the human population against the adverse effects of DON and its derivatives (Entry 28, Table 1) [98, 99]. In human Caco-2 cell monolayers and mice, GOS protect the intestinal barrier by maintaining the tight junction network and modulating inflammatory responses after a challenge with DON [98]. Furthermore, GOS stabilize the expression and cellular distribution of claudin 3 and suppress DON-induced synthesis and release of IL-8 [IL8/chemokine CXC motif ligand (CXCL8)] [99]. In mice, GOS prevent DON-induced mRNA overexpression of claudin 3 and CXCL8 homolog keratinocyte hemoattractant (Kc) (Cxcl1), as well as DON-induced morphologic defects [98]. Considering that the DON-induced alterations in the intestinal tract of mice resemble those in humans with chronic inflammatory diseases or regular exposure to DON, further studies should assess in greater detail the potential beneficial effects of GOS to support therapy aimed at prevention of toxin-induced inflammatory bowel diseases and related syndromes.

**Figure 4:**
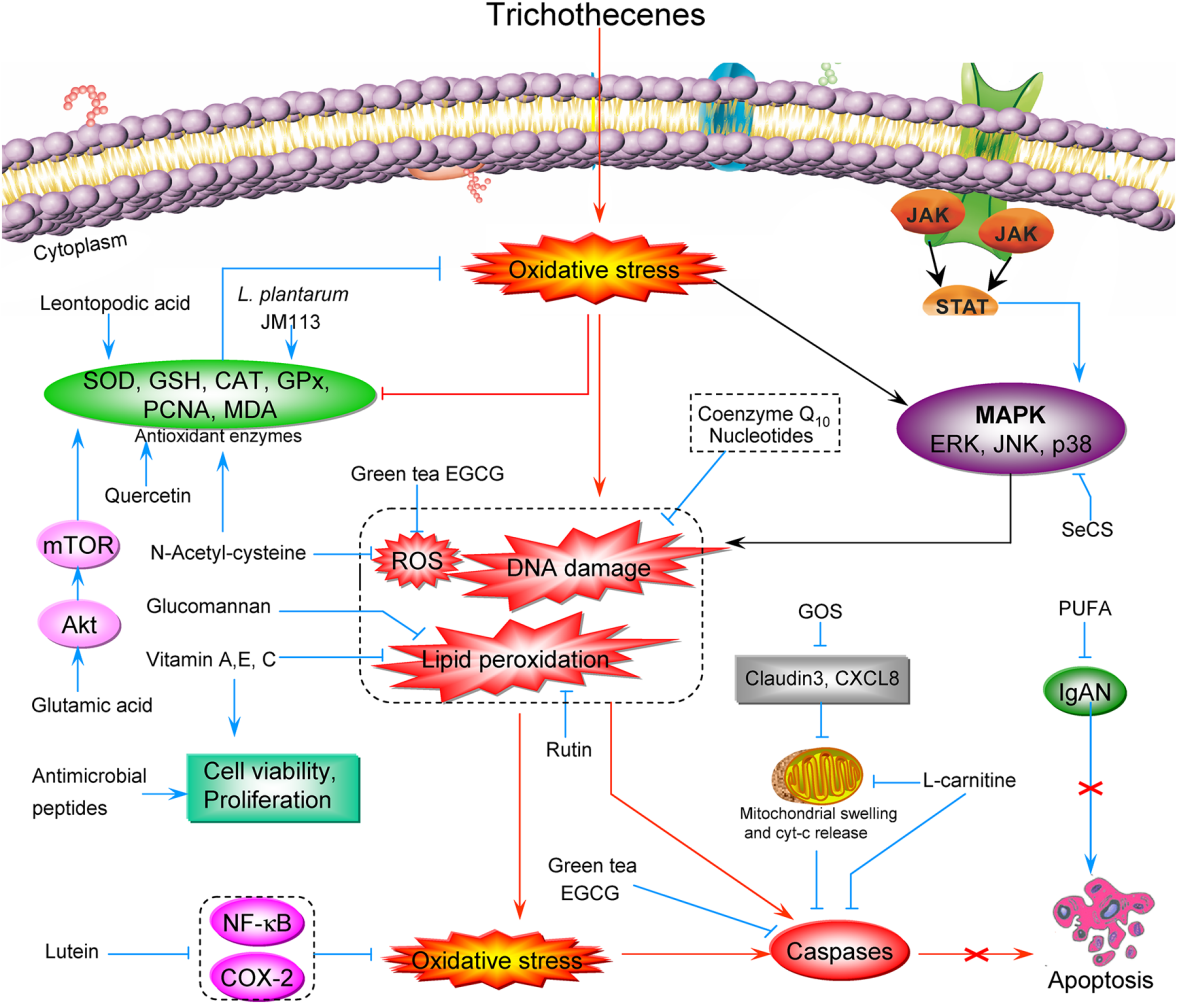
Schematic illustration of the preventive effects of various antioxidants on trichothecene-induced oxidative stress

In summary, many natural compounds exhibit anti-oxidative capacity against trichothecenes. As we described above, NAC and green tea EGCG inhibit ROS generation; coenzyme Q_10_ and nucleotides reduce DNA damage; and vitamins A, E, and C, glucomannnan, and rutin can reduce lipid peroxidation. On the other hand, other compounds target the antioxidant enzymes: quercetin, LA, and *L. plantarum* JM113 increase the activity of antioxidant enzymes such as SOD and CAT, thereby reducing oxidative stress and apoptosis. GOS prevent DON-induced overexpression of mRNAs encoding claudin 3 and CXCL8. L-carnitine not only diminishes oxidative stress, but also protects mitochondria against fatty acid stress and apoptosis by inhibiting mitochondrial swelling and Cyt-c release. In addition, L-carnitine and green tea EGCG inhibit caspase activity, thereby directly inhibiting cell apoptosis. Lutein combats DON-induced oxidative stress and downregulates expression of the inflammatory factors NF-κB and COX-2. SeCS can partly block trichothecene-induced chondrocyte apoptosis by decreasing MAPK signaling activity. A schematic illustration of the preventive effect of various antioxidants on trichothecene-induced oxidative stress is shown in Figure 4.

## OTHER DECONTAMINATION STRATEGIES

In addition to the antioxidant and detoxification agents described above, some decontamination methods are available. These approaches include bacterial and yeast biotransformation and degradation, as well as mycotoxin-binding agents. Because biological reactions offer a specific, irreversible, efficient, and environmentally friendly means of detoxification that leaves neither toxic residues nor any undesired by-products, microbial biotransformation of mycotoxins into non-toxic or low-toxin metabolites has been acknowledged as a promising approach [100]. The isolation and characterization of microorganisms that can biotransform mycotoxins is crucial for practical applications of biotechnology [101]. Based on the ability of lactic acid bacteria (LAB) to inhibit fungal development and remove multiple mycotoxins, including DON and aflatoxins, along with their Generally Recognized As Safe (GRAS) status and probiotic potential, these organisms are candidates for the biological control of fungi such as *F. graminearum* in crops or even during food production and processing [102, 103]. Increasing evidence shows that LABs could effectively remove trichothecenes T-2 and DON from cereals. For example, Franco et al. (2011) [103] evaluated the ability of different LAB strains to remove DON *in vitro*. All of the isolated strains and commercial cultures tested in that study inhibited fungal growth and decreased DON levels by 16.41–55.30%. Although the mechanism remains unclear, the detoxification capacity of LAB could be related to metabolic degradation or adsorption of trichothecenes by the bacterial cell wall [104]. In another study, five strains of LAB were tested for their ability to remove DON and T-2 toxin [105]. The capacity of *Lactobacillus plantarum* strain 102 (LP102) was the strongest after incubation at 37°C for 72 h. Recently, Zhao et al. (2016) [106] identified a microbial community, *Devosia sp.* ANSB714, which could degrade 97.34% DON after 24 h. In mice, a diet containing DON reduces growth performance and affects liver, kidney, and immune function. Addition of *Devosia sp*. ANSB714 to DON-contaminated diets eliminates these effects of DON. Therefore, detoxification with *Devosia sp.* ANSB714 could have an enormous impact in the livestock industry.

In addition to LAB, the yeast *Geotrichum candidum* also exhibited a strong ability to reduce T-2 toxin levels in cereals. For example, an interaction between *G. candidum* and *F. langsethiae* led to a drastic (93%) reduction in T-2 toxin production [107]. The mechanism remains unclear, but we suspect that the yeast might transform T-2 toxin to another compound or inhibit its production by the fungi. Indeed, McCormick et al. (2012) [108] showed that several yeast species can convert T-2 toxin to 3-acetyl-T-2 toxin, T-2-3-glucoside, and neosolaniol (NEO), which are much less toxic than the parent compound [1]. In addition, a mixture of inorganic (activated carbon) and organic (yeast cell wall) adsorbents contributed to *in vitro* removal of DON. The highest percentage of adsorption occurred when 2.0% activated carbon and yeast cell wall were present at a 30:70 ratio (>95.6%) and incubation continued for 30, 60, or 90 min [109]. Another study confirmed that yeast-based feed additives could reduce DON-induced oxidative stress in pigs [110].

Microbial detoxification represents an alternative approach to physical and chemical methods for detoxification of DON-contaminated grain. A recent study showed that a novel bacterium, *Devosia mutans* 17-2-E-8, was capable of transforming DON to a non-toxic stereoisomer, 3-epi-DON, under aerobic conditions, mild temperature, and neutral pH [40]. Moreover, the process was enzymatic in nature and had a high detoxification capacity. These observations suggest the possibility of using the isolated bacterium as a feed treatment to address DON contamination under empirical field conditions. Another trial was conducted to evaluate a feed additive containing epoxidase activity from a bacterium (Mycofix-S) as potential protection against the adverse effects of T-2 toxin in growing male broiler chickens [111]. The results showed that T-2 toxin at 2.5 ppm significantly decreased body weight gain and cumulative feed intake. However, the feed additive counteracted these adverse effects. Moreover, a significant decrease in amylase activity was observed in chickens receiving T-2 toxin, indicating that the additive also protected against duodenal lesions caused by T-2 toxin.

Several commercially available feed additives have the potential to reduce the toxicity of trichothecenes [112–114]. Some of these materials, such as clays, are being used as anti-caking agents to improve flow during feed processing, but they may also serve as mycotoxin-binding agents [110–115]. For example, in a study by Weaver et al. (2013) [110], the feed additives clay and dried yeast were tested to improve the growth and health of pigs chronically challenged with DON. Dietary DON tended to decrease average daily gain and altered the immune system by increasing the levels of monocytes and immunoglobulins. DON also caused tissue damage in the form of liver bile ductal hyperplasia and karyomegaly. However, the additives reduced the effects of DON on the immune system and liver, and also improved growth. Ozonation is another effective and rapid way to degrade DON in wheat, especially in wheat flour [116]. On the other hand, water-soluble DON poses a major threat as a potential organic pollutant to water environmental quality. In wet processing, soluble-toxin molecules can be extracted from the contaminated grain by DON, posing a potentially serious threat to the water environment. In a very recent study [117], ZnO/graphene monolayer hybrid with high photocatalytic performance was shown to effectively degrade DON in water (99% reduction). This work provided important inspiration for the development of graphene-based photocatalysts for use in applications related to trichothecene detoxification and environmental remediation.

These detoxifiers can be broadly categorized into two different classes, mycotoxin binders and mycotoxin modifiers/biotransforming agents. The latter class includes microbes such as bacteria, fungi, yeast, and enzymes that biologically transform the toxins into non-toxic metabolites. LAB, a promising candidate for the biological control of trichothecenes, may biotransform the toxins to less toxic products. In addition, some bacteria including *Devosia mutans* 17-2-E-8 and Mycofix-S can also biotransform DON into non-toxic compounds, thereby protecting against duodenal lesions caused by T-2 toxin. However, although some decontamination has already been reported, very few commercially available feed additives can reduce the toxicity of mycotoxins such as trichothecenes. Moreover, the biotransforming agents tested to date, such as LAB and yeast, are still limited in the laboratory. Future work should seek to develop methods that allow these agents to be used as real feed additives in the commercial market.

## CONCLUSIONS

Trichothecenes inhibit eukaryotic protein synthesis, and oxidative stress is one of their most important underlying toxic mechanisms. They are able to generate free radicals, including ROS, which induce lipid peroxidation that leads to changes in membrane integrity, cellular redox signaling, and cellular antioxidant status. DNA damage correlates with, but precedes, ROS generation and lipid peroxidation. The MAPK signaling pathway is induced by oxidative stress, which also induces caspase-mediated cellular apoptosis pathways. Thus, it is clear that oxidative stress plays important roles in the toxicity of trichothecenes. Notably, however, cells have their own antioxidant defenses against these toxins. The cellular effects of trichothecenes in relation to oxidative stress, as well as effective measures for combating their toxicity, should be addressed further in future studies.

Because oxidative stress plays critical roles in the toxicity of trichothecenes, researchers are trying to identify agents that effectively prevent trichothecene-induced oxidative stress and the associated immunotoxicity. Multiple agents, including vitamins, quercetin, selenium, glucomannan, amino acids, nucleotides, antimicrobial peptides, bacteria, polyunsaturated fatty acids, oligosaccharides, and some plant extracts, exert promising anti-oxidative effects against trichothecenes. Thus, these antioxidant agents should be considered as new toxin control strategies for trichothecenes. We conclude that these compounds inhibit oxidative stress via various mechanisms, e.g., diminishing ROS generation (NAC, EGCG), decreasing DNA damage (nucleotides and coenzyme Q_10_), reducing lipid peroxidation (vitamins A, C, and E, glucomannan, and rutin), increasing the activity of the antioxidant enzymes (LA, *L. plantarum* JM113, NAC, and quercetin), blocking the induction of MAPK signaling (SeCS), downregulating the expression of inflammatory genes NF-κB and COX-2 (lutein), inhibiting caspase activity (EGCG and L-carnitine), preventing mRNA overexpression of claudin 3 and CXCL8 (GOS), or inhibiting mitochondrial swelling and cyt-c release (L-carnitine).

Some of these compounds are also employed in the detoxification of other mycotxoins. For example, ochratoxin A (OTA), a widely spread mycotoxin [118], inhibits protein synthesis and energy production, and induces oxidative stress and DNA adduct formation, as well as apoptosis/necrosis and cell cycle arrest [119]. Similar to previous observations in trichothecenes, vitamins A, C, E, EGCG, and quercetin increase kidney cell proliferation, and decrease OTA mediated-ROS generation and DNA fragmentation [120–122]. Understanding these mechanisms of antioxidant agents will aid in the development of methods for detoxification of mycotoxins, as well as new toxin control strategies. In future studies, additional *in vivo* studies should be carried out to characterize their anti-oxidative activities in animals and humans.

As we discussed in the above chapters, the antioxidant defences induced by dietary supplementation would seem to provide a more reasonable and practical approach for the reduction of oxidative stress levels [123]. In addition, previous studies [123–125] have shown that a variety of proteins function as scavengers of superoxide and hydrogen peroxide, including catalase, glutathione peroxidase, thioredoxin, and the peroxiredoxin protein family. Together with these, a whole host of non-protein scavengers, including, but not limited to, intracellular ascorbate and glutathione [124] also reduce oxidative stress levels. However, the successful implementation of these approaches will probably require a much greater understanding of the pharmacological properties of many of these agents, including their rates of absorption, tissue distribution, metabolism, and the microenvironment in which they must act [123]. Interestingly, *Mycobacterium tuberculosis* (Mtb), a virulent and deadly bacterial species, has evolved strategies to counter ROS-mediated host defences by expressing enzymes that detoxify a variety of free radical species [126]. It is noteworthy that a novel multicomponent protein complex with both peroxidase and peroxynitrite reductase activity has been identified [127]. A future study should address the possibility of using such multicomponent protein complexes as antioxidant therapies against trichothecenes.

In addition to the antioxidant and detoxification agents described above, several decontamination methods exist, including bacterial and yeast biotransformation and degradation, as well as mycotoxin-binding agents. Because biological reactions offer a specific, irreversible, efficient, and environmentally friendly means of detoxification that leaves neither toxic residues nor any undesired by-products, microbial biotransformation of mycotoxins into non-toxic or less toxic metabolites has been acknowledged as a promising approach. Finally, we should not forget that various food processing methods including baking, boiling, frying, steaming, and extrusion cooking have some positive effects in the controlling of the contamination in foods [128, 129].

## Abbreviations

ATA: alimentary toxic aleukia
CAPs: composite antimicrobial peptides
CAT: catalase
mESCs: embryonic stem cells
CREB: cAMP response element-binding protein
CXCL8: IL8/chemokine CXC motif ligand
DHA: docosahexaenoic acid
DON: deoxynivalenol
EGCG: epigallocatechin 3-gallate
EPA: eicosapentaenoic acid
GH: growth hormone
GOS: galacto-oligosaccharides
GPx: glutathione peroxidase
GSH: glutathione
GSH-Px: glutathione peroxidase
GST: glutathione-S-transferase
HO-1: heme oxygenase-1
IgAN: IgA nephropathy
KBD: Kashin-Beck disease
LA: leontopodic acid
LAB: lactic acid bacteria
LGG: *L. rhamnosus* GG
MAPK: mitogen-activated protein kinases
MDA: malondialdehyde
NAC: N-Acetyl-cysteine
NEO: neosolaniol
NO: nitric oxide
PCNA: proliferating cell nuclear antigen
PKR: RNA-activated protein kinase R
PUFA: polyunsaturated fatty acid
ROS: reactive oxygen species
RSR: ribotoxic stress response
SeCS: selenium chondroitin sulfate
SOD: superoxidae dismutase
TAS: total antioxidant status
TBARS: thiobarbituric acid reactive substances
ΔΨm: mitochondrial membrane potential.
